# An Unusual Case of Immune Complex-Mediated Membranoproliferative Glomerulonephritis as Renal Manifestation of Idiopathic Hypereosinophilic Syndrome: A Case Report and Literature Review

**DOI:** 10.3390/medicines11060013

**Published:** 2024-06-02

**Authors:** Michael Cieza-Terrones, José C. De La Flor, Christian Requejo, Daniel Villa, Jacqueline Apaza, Pablo Rodríguez-Doyágüez, Rocío Zamora, Carmen Asato-Higa, David Rivera-Estrella, Antonio Carrasco-Yalán

**Affiliations:** 1 Department of Nephrology, Hospital Cayetano Heredia, Faculty of Medicine, Peruana Cayetano Heredia University, Lima 15002, Peru; michael.cieza@upch.pe (M.C.-T.); christian.requejo@upch.pe (C.R.); david.rivera@upch.pe (D.R.-E.); 2 Department of Nephrology, Hospital Central Defense Gomez Ulla, 280467 Madrid, Spain; 3 Faculty of Medicine, Alcala de Henares University, 28805 Madrid, Spain; 4 Department of Nephrology, Clínica Universidad de Navarra, 31008 Navarra, Spain; devillah@unav.es; 5 Department of Nephrology, Hospital Rey Juan Carlos, 28933 Madrid, Spain; jacqueline.apaza@hospitalreyjuancarlos.es; 6 Department of Nephrology, Guadalajara Center Dialysis, AVERICUM, 19003 Guadalajara, Spain; pablo.rodriguez@avericum.com; 7 Department of Nephrology, Hospital Universitario General Villalba, 28400 Madrid, Spain; rocio.zamora@hgvillalba.es; 8 Anatomic Pathology Department, PATOLOGAS AS SAC, Lima 15082, Peru; patologas_as@hotmail.com; 9 Postgraduate School, Faculty of Medicine, Universidad Nacional Mayor de San Marcos, Lima 15081, Peru; antonio.carrasco@unmsm.edu.pe

**Keywords:** idiopathic hypereosinophilic syndrome, immune complex-mediated membranoproliferative glomerulonephritis, nephrotic syndrome, acute kidney injury

## Abstract

Background: Idiopathic hypereosinophilic syndrome (IHES) is a disorder characterized by abnormal and persistent peripheral blood hypereosinophilia (eosinophil count ≥ 1.5 × 10^9^/L and ≥10% eosinophils) with duration ≥ 6 months, associated organ damage, and/or dysfunction attributable to tissue eosinophilic infiltrate of unknown cause. IHES affects different organs such as the heart, lungs, nervous system, and skin, with renal involvement being rare in this condition. Case Presentation: We present a case of a young patient with IHES and immune complex-mediated membranoproliferative glomerulonephritis with nephrotic syndrome, as a rare renal manifestation. We discuss the clinical, analytical, and histopathologic renal and hematologic features, comparing them with other reported cases in the literature.

## 1. Introduction

Hypereosinophilic syndrome (HES) is a rare hematologic disorder with a prevalence ranging from 0.36 to 6.3 per 100,000 habitants [1]. HES can be idiopathic (IHES) or of undetermined significance, hereditary, primary (clonal/neoplastic), or secondary (reactive). IHES is defined according to the World Health Organization [2], and the International Consensus Classification [3], on eosinophilic disorders as abnormal persistent peripheral blood hypereosinophilia (eosinophil count ≥ 1.5 × 10^9^/L and ≥10% eosinophils) with duration ≥ 6 months, associated organ damage, and/or dysfunction attributable to tissue eosinophilic infiltrate of unknown cause, without evidence of a well-defined reactive autoimmune disease, genetic, allergic reactions, infectious disease, or neoplastic condition/disorder underlying the hypereosinophilia, with bone marrow biopsy (BMB) morphologically within normal limits without any molecular genetic clonal abnormality, with the exception of clonal hematopoiesis of undetermined potential (CHIP). It is also necessary to discard hypereosinophilic syndrome, a lymphocytic variant. IHES is more common in men and usually occurs between 20 and 50 years of age [2]. IHES affects different organs such as the heart, lungs, nervous system, and skin. Renal involvement is rare in this disease, and the prevalence could range from 7 to 36% [4,5]. Due to the lack of studies, data on renal damage come from case reports and are classified according to the area of eosinophilic infiltration: glomerular, tubulointerstitial, vascular involvement, and/or electrolyte disturbances. Characteristic manifestations described were arterial hypertension, acute kidney injury (AKI) and/or chronic kidney disease (CKD), electrolyte disturbances, microhematuria, and variable proteinuria. Histopathologic patterns described were as follows: eosinophilic interstitial nephritis, membranous nephropathy (MN), crescent glomerulonephritis (CGN), focal segmental glomerulosclerosis (FSGN), immunotactoid glomerulonephritis (IGN), and renal thrombotic microangiopathy (TMA) [6,7]. Two possible pathophysiological mechanisms of renal involvement in IHES are considered, one of them is ischemic renal damage secondary to mural thrombus of cardiac origin, mediated by eosinophilic cytotoxicity and leading to a state of pro-coagulation and vascular dysfunction (mainly the cases of TMA), and the other is by a direct eosinophilic cytotoxic effect due to eosinophilic mass cell infiltrates [7,8,9].

IHES has a poor prognosis if left untreated, with cardiac involvement being the most significant marker of severity and the most frequent cause of mortality [5]. The first line of treatment is corticosteroids, which aim to reduce eosinophilia; however, if this does not occur, an additional immunosuppressive or biologic medication, such as cyclophosphamide or imatinib, respectively, could be used as a second-line therapy. Additional agents that have been used to rapidly lower counts in steroid-refractory patients include hydroxyurea, vincristine, and interferon alpha, which are less used. Nowadays, the availability of other biologics such as mepolizumab provides more therapeutic alternatives. Unfortunately, there are currently no predictors or profiles of involvement to select an ideal biologic drug, so the response to many of the mentioned drugs is variable and there is no accurate data describing the efficacy of eosinophil depletion. In most cases, recovery of renal function and reduction in proteinuria are accompanied by the resolution of hematologic disease [6,10].

We report the case of a young patient with IHES and immune complex-mediated membranoproliferative glomerulonephritis (IC-MPGN) with nephrotic syndrome, which is a rare renal manifestation, and discuss the clinical and renal histopathologic features together with similar cases in the literature.

## 2. Case Presentation

Our patient is a 23-year-old Caucasian male, with no significant medical history. He went to the emergency room due to general malaise, asthenia, a fever of 39 °C, and dull pain in the shoulders and anterior chest region after 6 weeks of evolution. Five weeks earlier, he began to have edema that progressed to the abdominal wall, hyporexia, frothy, and choluric urine, and a dry cough. The physical examination revealed a respiratory rate of 24 breaths/min; uncontrolled arterial hypertension (160/90 mmHg); an oxygen saturation of 96% while breathing room air; and a body temperature of 37.8 °C. He had mild pallor of skin and mucous, pulmonary rales, and at least three peripheral pitting edema in the lower limbs without purpuric lesions. There was no lymph node involvement, splenomegaly, arthralgia, bone pain, or Raynaud’s phenomenon. He denied a history of asthma, and no clinical signs of this disease were found on examination. He denied episodes of angioedema or oral ulcers. Complementary tests showed the following: a hemoglobin of 10 g/dL, leukocytes 31,500/L with 60% eosinophils (18,900/L), and platelets 416,000/L.; normal haptoglobin; reticulocytes 2.53%, blood smear without abnormal cells; negative Coombs test; and normal ADAMTS-13 activity. A renal function test showed serum creatinine (SCr) of 1.21 mg/dL (being the previous basal SCr values of 0.7 mg/dL), an estimated glomerular filtration rate (eGFR) of 83.9 mL/min/1.73 m^2^, calculated based on Chronic Kidney Disease Epidemiology (CKD-EPI) 2021 creatinine equation; and the urinalysis showed a macrohematuria of 200 red blood cells per high-power field (RBC/HPF) with dysmorphic cells and urine protein of 569 mg/dL. Hypoalbuminemia (2.79 g/dL) without hypercholesterolemia was present. The 24 h urine protein excretion was 11 g/day. Serum protein immunofixation/electrophoresis (IFE) revealed a polyclonal distribution. Urine IFE was negative to monoclonal bands. Hypergammaglobulinemia IgG (1700 mg/dL), free light chain kappa (FLCκ), and lambda (FLCλ) of 15 mg/dL and 20 mg/l, respectively, were found, including a Kappa/Lambda ratio of 0.75. The total IgE level was 12,660 IU/mL (normal < 200 IU/mL). Complements C3 and C4 were normal. We requested a complete microbiological stool and blood study to discard hypereosinophilia secondary to infections. The immunofluorescence test for hydatidosis and toxoplasmosis was negative. The stool study also failed to demonstrate eggs, cysts, or parasites. The tuberculin test was negative. Blood and urine cultures were negative. Serology was negative for syphilis, herpesvirus 2, human immunodeficiency virus (HIV), hepatitis B (HBV), hepatitis C (HCV), Epstein-Bar virus (EBV), herpes virus 1, CMV, and COVID-19. The immunological study for Antinuclear antibody (ANA), anti-double-stranded (DNA) antibodies, Anti-Smith (Sm) antibodies, Anti-ribonucleoprotein (RNP) antibodies, perinuclear anti-neutrophil cytoplasmic antibody (p-ANCA), c-ANCA, rheumatoid factor (RF), anti-Ro, anti-La antibodies, and serum cryoglobulins were negatives (Table 1). Allergy skin tests for pollen, pet dander, dust mites, drugs, and food were negative. Abdominal sonography revealed normal-sized kidneys. Our patient’s chest X-ray showed clear lungs, a healthy heart, and a clearly demarcated thoracic cavity. Electrocardiogram (ECG) and echocardiogram were normal. In order to discard an occult neoplasm, gastrointestinal studies, scintigraphy, thyroid function study, and thoraco-abdominal-pelvic computed tomography (CT) were performed, all of which were normal.

**Figure 1:**
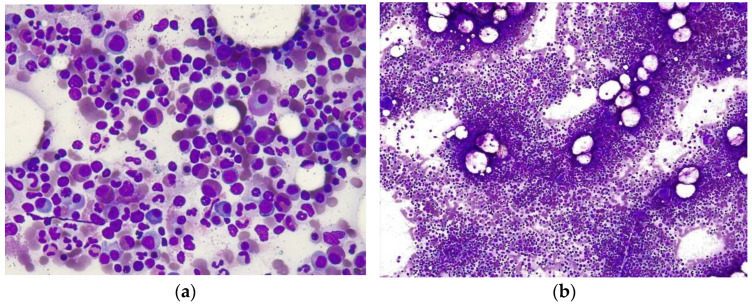
(**a**) Bone marrow aspiration showed moderate hyperplasia of the myeloid lineage, most of which consisted of mature eosinophils, without signs of dysplasia, accounting for over 40% of the total mononuclear cells. (**b**) Bone marrow biopsy shows a marked expansion of mature eosinophils.

A bone marrow aspirate (BMA) was performed, revealing a cellularity of 65%, with a marked increase in eosinophils (40%) and immature eosinophil precursors with a myelo-erythroid ratio (MER) of 5:1, and no fibrosis and no mast cells were observed (Figure 1a). BMB confirmed these findings, with a cellularity of 70% eosinophils, without evidence of myeloproliferative or lymphoproliferative disorder, multiple myeloma, or metastatic neoplasm (Figure 1b). The bone marrow cytometry was as follows: in the neutrophil granulocyte compartment (30.74%), a continuous maturation pattern was observed (CD11b vs. CD13), with dysplastic phenotypic characteristics (CD16 vs. CD13). The eosinophilic population was 51.14%. For the tumor determination of the genes studied, AmpliSeq technology was used in combination with NGS (Next Generation Sequencing) sequencing from Illumina (San Diego, CA, USA) to determine variants in genes associated with myeloid disease. No pathological alterations were found in the following genes: *ABL1*, *ALK*, *ASXL1*, *BAALC*, *BLC2*, *BCOR*, *BRAF*, *CALR*, *CBL*, *CCND1*, *CEBPA*, *CREBBP*, *CSF3R*, *DMMT3A*, *EIF2B1*, *EGFR*, *ETV6*, *EZH2*, *FBXW2*, *FGFR1*, *FGFR2*, *FLT3A*, *FUS*, *GATA2*, *HMGA2*, *HRAS*, *IDH1*, *IDH2*, *IKZF1*, *JAK2*, *KIT*, *KMT2A* (*MLL*), *KRAS*, *MECOM*, *MET*, *MLLT10*, *MLLT3*, *MPL*, *MYBL1*, *MYC*, *MYD88*, *MYH11*, *NF1*, *NPM1*, *NRAS*, *NTRK3*, *NUP214*, *PDGFRA*, *PDGFRB*, *PHF6*, *PSMB2*, *PRPF8*, *PTPN11*, *PUM1*, *RARA*, *RB1*, *RBM12*, *RUNX1*, *SETBP1*, *SH2B3*, *SF3B1*, *SMC1A*, *SRSF2*, *STAG2*, *TCF3*, *TET2*, *TFE3*, *TP53*, *TRIM27*, *U2AF1*, *WT1*, and *ZRSR2*. RT-PCR was negative for the FIP1L1-PDGFRA transcript. The subcutaneous fat biopsy showed negative Congo red staining. A presumptive diagnosis of HES was made based on presenting symptoms, analytical data, imaging studies, and BMB. The investigation of secondary causes, including immunological tests, blood cultures, urine cultures, stool cultures for parasites, and infectious serology, revealed nothing, as did urine drug screening and allergy testing, establishing the diagnosis of IHES.

**Figure 2:**
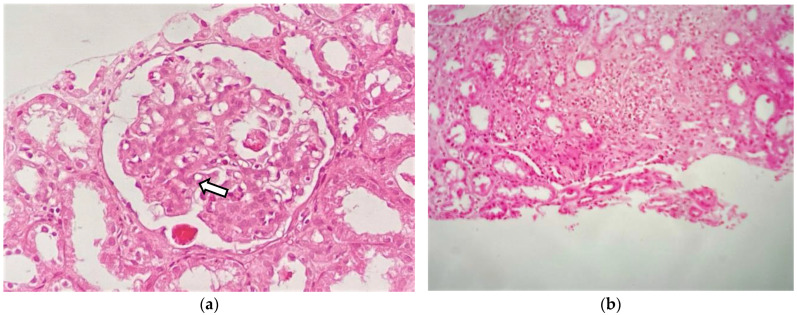
Renal biopsy: (**a**) light microscopy shows a glomerulus with moderate mesangial hypercellularity (white arrow) (H-E staining; original magnification ×400); (**b**) interstitial inflammatory infiltrate composed of lymphocytes and eosinophils (H-E staining; original magnification ×200).

**Figure 3:**
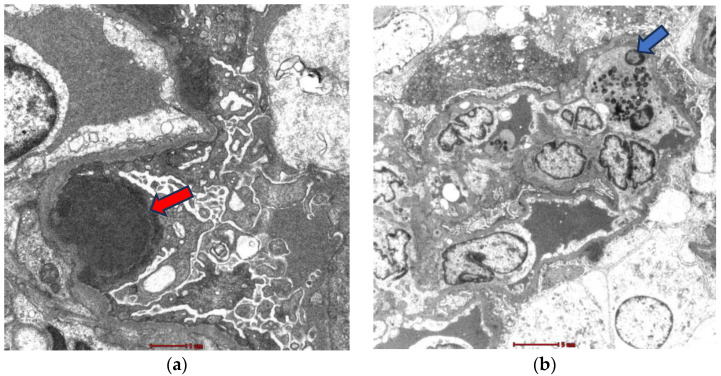
(**a**) Ultrastructural study shows subepithelial electrodense deposits in the basement membrane (red arrow) along with fusion of the epithelial cell foot processes; (**b**) endocapillary proliferation with the presence of intracapillary eosinophils (blue arrow).

**Figure 4:**
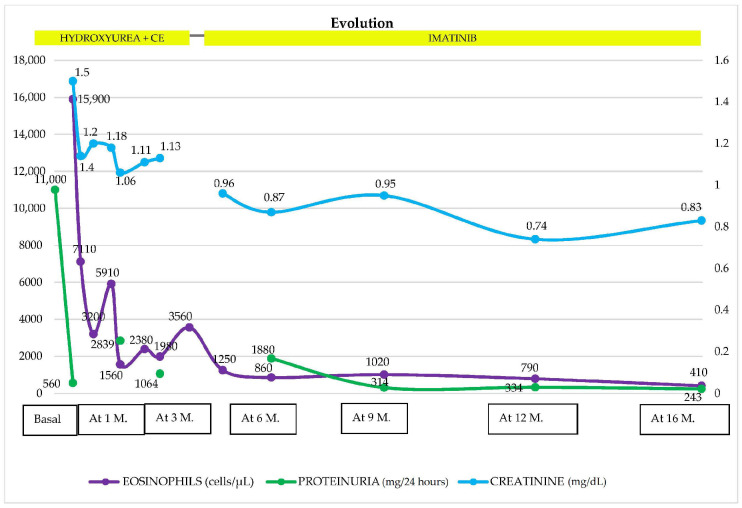
Evolution of hematological and renal parameters at follow-up, 16 months after the start of treatment. GC: glucocorticoids, M: month, Proteinuria (mg/g).

Given the renal involvement, characterized by a nephrotic/nephritic syndrome, a renal biopsy (RB) was performed. Light microscopy (LM) showed 18 glomeruli, 1 of which (5.5%) was globally sclerotic. The rest showed lobular accentuation, expansion, and proliferation of mesangial cells (2+), the occasional presence of polynuclear cells in capillaries, 2–5 eosinophils, and in addition, thickening and segmental unfolding of capillary loops, and 3 of 18 glomeruli showed cellular crescents and red blood cells in capillary lumens (1+). Tubules showed turbid degeneration and hematic casts. The interstitium showed a lymphoid infiltrate and mild eosinophils. Arteries and arterioles were unremarkable (Figure 2a,b). Congo red and thioflavin staining were negative. The frozen tissue immunofluorescence (IF-F) study was negative for IgG, IgA, IgM, C3, C4, LCκ, LCλ and fibrinogen. IF on pronase-digested paraffin-embedded (IF-P) sections was not performed in our case. The immunohistochemical staining study was negative for Anti-Phospholipase A2 Receptor Antibody (Anti-PLA2R), IgG4 and C4d. Electron microscopy (EM) revealed glomerular subepithelial granular 1+ glomerular deposits (humps) distributed in a disordered manner and of heterogeneous size, a basement membrane of capillary loops with sporadic thickened segments, endothelial cells with preserved fenestrations, no tubule reticular inclusions, and abundant cells in the lumen of the capillaries (eosinophils, neutrophils, and lymphomononuclear cells). Diffuse pedicellular effacement in podocytes and focal hyperplasia, increased mesangial matrix, tubular atrophy, and an interstitium with increased collagen and the presence of leukocytes and lymphomononuclear cells were observed. The vessels did not present significant alterations (Figure 3a,b). Based on the clinical course, and the analytical and histological findings of LM, IF, and EM, the diagnosis was diffuse membranoproliferative glomerulonephritis (MPGN) due to deposition of unorganized immune complexes, secondary IHES. In view of the above results, and in the presence of a massive nephrotic proteinuria with AKI, it was decided to start treatment with glucocorticoids (GC), at a dose of 1 mg/kg/day of prednisone (PDN) with progressive tapering after 2 weeks and discontinuation at 12 weeks and hydroxyurea, which was administered initially at 2 g/day for 7 days. The use of both drugs resulted in a favorable evolution characterized by a 50% reduction in eosinophilia concentration as well as a significant decrease in proteinuria and creatinine (Figure 4). After this improvement, the hydroxyurea dose was adjusted to 1.5 g/day for 10 days and subsequently 1 g/day for 14 days. However, an increase in the eosinophilia concentration was observed with this reduction, leading to increasing the dose to 2 g/day again. This adjustment achieved an adequate response for 14 days. Subsequently, the dose was reduced to 1 g and maintained for 90 days. However, in the third month after the start of treatment, the patient presented a new rebound in eosinophil values associated with the persistence of residual proteinuria of 1064 mg/g. In view of this situation, and in order to avoid the toxicity of hydroxyurea and GC minimization, it was decided to switch to imatinib at 400 mg/day. The patient responded very well to this new approach. At the 12-month follow-up, a reduction in the eosinophil concentration to below 800/L was noted, with creatinine levels of 0.74 mg/dL and 24 h urine protein of 334 mg (Figure 4). Response to treatment was favorable, evidencing a positive evolution in his clinical condition. The results coincided with a significant decrease in IgE concentration to values of 81.7 IU/mL (normal < 200 IU/mL). It was only necessary to add, as an antiproteinuric measure, a renin-angiotensin aldosterone system (RAAS) blockade; it was not necessary to add mineralocorticoid receptor antagonists (MRAs) or sodium-glucose transporter protein 2 inhibitors (SGLT2i), due to their decrease in proteinuria to non-nephrotic ranges. At the 16-month follow-up, he continued to have a normal eosinophil count, proteinuria, and renal function.

## 3. Discussion

We present the case of a young male patient with IHES and renal involvement. The association between glomerulonephritis and IHES is rare, which makes this case a valuable contribution to the medical field. To make the diagnosis of IHES, it is essential to discard hereditary, primary, and secondary causes of HES. In our case, neoplastic diseases, parasitosis, and collagenosis were reasonably discarded, being interpreted as an IHES without cardiac involvement (which is usually early and the leading cause of death) and associated with IC-MPGN as a renal manifestation. This exclusion process is essential to ensure an accurate diagnosis and to allow an adequate and individualized therapeutic approach for each patient [11]. Our patient presented a remarkable leukocytosis, with a significant predominance of eosinophils (60%). These analytical findings provided a solid basis for the diagnosis of HES. The most described definition of HES is based on a peripheral blood eosinophil count greater than 1.5 × 10^9^/L accompanied by organ or tissue damage [12]. However, it is important to keep in mind that some patients with HE may have organ or tissue damage with a lower eosinophil count or have counts above 1.5 × 10^9^/L without evidence of organ damage. These scenarios should be considered in the evaluation of a patient with HES [13]. The organs most frequently affected by IHES show a variety of clinical manifestations. Regarding skin involvement, angioedema, urticaria, or other types of skin lesions may be present, while for cardiac involvement, the clinical manifestations may be expressed as cardiomyopathy, valvular damage, and heart failure, the latter being the severity of the picture. At the pulmonary level, bronchial hyperreactivity may be present with a chronic course and evolve toward pulmonary fibrosis. The nervous system may present as encephalopathy or neuropathy. Gastrointestinal involvement may cause symptoms such as abdominal pain or oral intolerance [14]. Another clinical disturbance in IHES is thrombocytosis, with an incidence described between 4% and 24% of cases [15,16]. The presence of venous and/or arterial thromboembolic events increases mortality. Aukstuolis K. et al. [17] hypothesized that the interaction between platelets and eosinophils favors the prothrombotic state in these patients.

Renal involvement in IHES is rare, and its clinical spectrum is broad [18]. Shehwaro et al. [7] performed a mini-review on the renal involvement in IHES, addressing the various manifestations involving glomerular, tubulointerstitial, vascular, and electrolyte disturbances. In the glomerular setting, cases characterized by the presence of proteinuria or a marked significant deterioration in GFR were identified. On the other hand, tubular manifestations included tubulointerstitial nephritis with eosinophil infiltration. In addition, vascular manifestations such as renal TMA were highlighted. Electrolyte disturbances are also described, mainly malignant hypercalcemia and renal hypouricemia. With regard to the first, the cause that triggers it is not known. Proposed mechanisms include bone destruction by an expansion and/or infiltration of an eosinophilic cell mass with a consequent mobilization of calcium (bone resorption), production of hypercalcemic humoral substances or local inflammatory cytokines such as interleukine-1 (IL-1), tumor necrosis factor, and IL-5 [7]. Renal hypouricemia was described in a case report of a patient with IHES related to a transient proximal tubule defect that resolved after GC treatment with a decreased hypereosinophilia concentration [19]. In addition, a case of crystalluria (Charcot–Leyden Crystals) and AKI in a patient with IHES was also described [20]. In our case, the patient presented AKI and nephrotic/nephritic syndrome as renal manifestations. Glomerular involvement associated with IHES usually manifests as a nephrotic syndrome in most of the case reports reported in the literature. Perez-Perez et al. [21] describe the case of a 31-year-old female patient with IHES-associated MN. This patient debuted with nephrotic syndrome, and although the response to GC managed to reverse the hypereosinophilia, proteinuria persisted in the nephrotic range. On the other hand, Choi et al. [22] reported a case of an IGN associated with IHES in an 18-year-old patient; the patient was asymptomatic, and the HE and altered renal function was an incidental finding. Likewise, Bulucu et al. [23] described the case of a 40-year-old patient with renal involvement and IHES, whose RB showed glomerular sclerosis with periglomerular fibrosis, mild mesangial proliferation, and infiltration of eosinophils, with deposits of IgG, IgM, IgA, and C3. On other occasions, as demonstrated by Navarro et al. [24], renal involvement may be the first manifestation of IHES. This author found severe chronic tubulointerstitial nephritis and marked eosinophilia in the RB of a 73-year-old patient. In addition, Ni H-F et al. [25] reported the case of a 25-year-old patient with nephrotic syndrome and IHES, with a good response to GC. Curras-Martin et al. [26] described the case of a 63-year-old patient with renal lesions associated with HES. RB revealed findings of glomerular and vascular thrombotic microangiopathy, together with interstitial fibrosis and inflammation with the presence of focal eosinophils. Dong et al. [6] described the largest case series of 18 patients with IHES and renal involvement, of whom 15 underwent RB. Among the findings, various conditions were identified, including MPGN, minimal change disease (MCD), mesangial proliferative nephritis, IgA nephropathy (IgAN), MN, chronic interstitial nephritis, and FSGN. Most patients had nephrotic syndrome, and some had elevated creatinine levels. Patients ranged in age from 19 to 67 years, with a mean of 36 years. In these cases, the presence of an eosinophilic infiltration in the renal interstitium and glomeruli in LM was noteworthy. In addition, IF-F showed deposits of IgG, IgA, IgM, and C3 in capillary loops and mesangial areas. As a renal histologic manifestation, most of the RB of these patients showed eosinophilic infiltration, both in the interstitial and in the glomeruli.

There is no specific systemic clinical manifestation associated with IHES and renal involvement and, in some cases, it can be asymptomatic as shown by Navarro et al. [24]. Our patient presented general malaise, a fever, edema, fatigue, hyporexia, and a cough, coinciding with some other reported cases. Our patient did not present cardiac involvement, despite the heart being a frequently affected organ, as described in the mini-review by Shehwaro et al. [7] and in the case report by Curras-Martin et al. [26]. Laboratory results showed anemia, also present in the report by Curras-Martin et al. [26] and in almost half of the patients described by Dong et al. [6]. In the reports of Ni H-F et al. [25] and Curras-Martin et al. [26], and in our case as well, there was a predominance of IgE, with it not being the same as the rest of the immunoglobulins. While we did not find a clear reason for its elevation, and we discarded secondary causes of IHES at the time, what is striking is that the decrease in IgE levels coincided with the positive renal and hematologic response in our patient. We consider it as a marker of clinical response to treatment in our case.

The LM of our patient showed mesangial expansion with mesangial cell proliferation and endocapillary hypercellularity with the presence of neutrophils and eosinophils in the capillary lumens. The presence of double contours and cellular crescents was also identified. In the interstitium, we observed a patchy inflammatory infiltrate composed of lymphocytes and eosinophils. The case reported by Ni H-F et al. [25] also described mesangial proliferation and eosinophilic infiltration in the tubulointerstitial, with IgM deposits. Curras-Martin´s case report [26] and Dong´s case series [6] reported the presence of eosinophil infiltration in the interstitium, and the latter 73% of patients had an infiltrate with the focal distribution. According to the studies reviewed, the presence of eosinophils in the glomerulus and cellular crescents is not common, as described by Dong´s case series [6].

Regarding IF-F, in our case, the tissue was negative for IgG, IgM, IgA, C3, C1q, LCκ, LCλ, and fibrinogen. This differs from other case reports [6,22,23,25], which showed positivity for IgG, C3, and fibrinogen; IgG, IgM, and IgA; C3, IgM, and IgG; IgA, IgM, and C3 in IF, respectively. Additionally, in the report by Ni H-F et al. [25], epimembranous IgM deposits were documented, whereas those described by Dong et al. [6] presented a histopathologic pattern of MPGN with Igs in the glomerular capillary loops and mesangial areas, so the presence of deposits on immunofluorescence is to be expected. To have a more complete study of the case, it would have been ideal to perform IF on formalin-fixed, paraffin-embedded tissue. Das N. et al. [27] conclude that this technique can increase the sensitivity and specificity of detecting immunoglobulins and complement deposits in RB. Unfortunately, we do not have access to this technique in our hospital.

The EM study, in our case, showed subepithelial electron-dense deposits associated with an irregular increase in the thickening of the glomerular basement membrane and extensive effacement of podocyte foot processes. Intracapillary inflammatory cells were observed. There are few case reports comparing these findings. The case series of Dong et al. [6] had subepithelial deposits, and in all these samples, immunofluorescence was positive for at least IgG. Effacement of the podocyte process was described in most of them with a reactive immunofluorescence description as well. On the contrary, in our case, there was probably also the presence of Ig deposits in the subepithelial space, and the technique performed could not identify them. IF-P sections could have contributed to completing the IF study. The presence of intracapillary lymphocytes and eosinophils was to be expected due to the nature of HES and correlates with reports of other cases.

Early treatment reduces morbidity and mortality and prevents complications [28,29]. The therapeutic objectives are to reduce the number of eosinophils and prevent organ damage and thromboembolic episodes. GC at doses of 0.5–1 mg/kg/day is usually the first-line treatment, but the dose should be lowered as soon as there is any evidence of response. Refractoriness occurs in 15–20% of patients, to whom cytotoxic agents (CA) are subsequently associated to achieve their reduction or definitive suppression. CA, such as hydroxyurea or cyclophosphamide (CTX), are also indicated in association with GC in patients with rapid disease progression [30]. Dahabreh et al. [31] conducted a prospective study, which showed that initial combination therapy with PDN (1 mg/kg/day) and hydroxyurea (2 g) controlled disease in 15 of 15 patients; remission was maintained with hydroxyurea alone (0.5–1 g/d) after progressive tapering of both compounds. In the case series of Dong et al. [6], all 18 patients were treated with oral GC or combined immunosuppressant therapy (7 patients with Tripterygium glycosides, 1 patient with CTX, and 1 patient with Tacrolimus). On the other hand, refractory cases could benefit from treatment with hydroxyurea, chlorambucil, or interferon-α and, in selected cases, mainly with myeloproliferative features, tyrosine kinase inhibitors (TKI) (imatinib), and monoclonal antibodies (anti-CD52, such as alemtuzumab, or anti-IL-5, such as mepolizumab) could play a role [28,29]. Our patient presented a good renal and hematologic response. Although initially, the treatment with GC and hydroxyurea was good, due to the rebound in the eosinophilia concentration, we decided to switch to imatinib for availability instead of mepolizumab (a drug with which we also had no experience), and with proper monitoring, our patient did not present any complications, keeping him currently in remission. These agents, commonly used in myeloproliferative disorders, have demonstrated efficacy in cases of IHES. An IHES case must be carefully evaluated, and other therapies besides GC as a frontline treatment must be considered, in cases of defined organ damage. TKI, such as imatinib, can provide rapid control of IHES with very low, if any, toxicity [32]. Our patient’s clinical and laboratory improvement after treatment supports its use in this setting.

## 4. Conclusions

This case shows IC-MPGN as a rare renal manifestation of IHES. The initial approach is based on excluding other etiologies and underscoring the significance of considering less common diagnoses in atypical presentations. The condition is associated with a high mortality rate, primarily due to delayed diagnosis; however, most patients respond favorably to first- and/or second-line therapies. A multidisciplinary strategy is crucial to prevent significant complications in patients with systemic renal disease.

## Figures and Tables

**Table 1:** Laboratory findings on admission.

		Normal Range—Units
WBC	31.5	4–10 × 10^3^/µL
Eosinophils	18.9	0–0.5 × 10^3^/µL
Hemoglobin (Hb)	10.8	12–16 g/dL
Platelet count (Plt)	416 × 10^3^	150–450 × 10^3^/µL
Reticulocytes count	2.53 %	2–4%
Lactate dehydrogenase (LDH)	174.1	135–214 IU/L
Total bilirubin	0.29	0.1–1 mg/dL
Total protein (TP)	8.84	6.4–8.7 g/dL
Serum Albumin	2.79	3–5.5 g/dL
Serum Iron	61	60–170 mcg/dL
Transferrin saturation	28.80%	20–50%
Ferritin	421.6	24–336 mcg/L
AST	18	5–32 IU/L
ALT	13	5–33 IU/L
Urea	65	17–60 mg/dL
Creatinine	1.21	0.7–1.2 mg/dL
Na+	138	135–145 mmol/L
K+	4.8	3.5–5.5 mmol/L
Cl-	103	95–110 mmol/L
C-Reactive protein	12	0.1–0.5 mg/dL
ESR	96	<15 mm/h
RF	Negative	<15 IU/mL
Parasitology Stool	Negative	NA
C3	98	90–180 mg/dL
C4	16	10–40 mg/dL
IgG	1700	800–1600 mg/dL
IgA	285	70–400 mg/dL
IgM	165	90–180 mg/dL
IgE	12,660	<200 UI/mL
β2-microglobulin	4.08	0–20 mg/dL
ANA, Antids-DNA, ANCA, and cryoglobulin	Negative	NA
Urine red blood cells	200	/HPF
24 h urine total protein excretion	11	<0.15 g/24 h
SPEP/SIFE	Polyclonal	NA
UPEP/UIFE	Negative	NA
FLC κ	15	4.90–13.70 mg/L
FLC λ	20	7.60–19.50 mg/L
FLC κ/λ	0.75	0.27–1.67

NA: not applicable, WBC: White blood cells, GOT: Glutamate-Oxaloacetate Transaminase, GPT: glutamate pyruvate transaminase, Na: Sodium serum, K: Potassium serum, Cl: Chloride serum, CRP: C-reactive protein, ESR: erythrocyte sedimentation rate, C3: Complement 3, C4: Complement 4, RF: rheumatoid factor, ANA: Antinuclear antibody, Antids-DNA: anti-double stranded DNA antibody, ANCA: anti-neutrophil cytoplasmic autoantibody, Ig: Immunoglobulin, SPEP: Serum protein electrophoresis, SIFE: Serum immunofixation electrophoresis, UPEP/UIFE: urine protein electrophoresis/urine immunofixation electrophoresis, FLC: free light chain, κ: kappa, λ: lambda, HPF: high-power field.

## Data Availability

No new data were created or analyzed in this study. The data used to support the findings of this study are available from the corresponding author on request (contact J.D.F., josedelaflor81@yahoo.com, jflomer@mde.es).
